# Clinical and genetic spectrum of GSD type 6 in Korea

**DOI:** 10.1186/s13023-023-02750-1

**Published:** 2023-06-01

**Authors:** Jong Woo Hahn, Heerah Lee, Moon Woo Seong, Gyeong Hoon Kang, Jin Soo Moon, Jae Sung Ko

**Affiliations:** 1 Department of Pediatrics, Seoul National University College of Medicine, Seoul, Korea; 2 Department of Pediatrics, Seoul National University Bundang Hospital, Seongnam, Gyeonggi- do Korea; 3 Department of Laboratory, Seoul National University College of Medicine, Seoul, Korea; 4 Department of Pathology, Seoul National University College of Medicine, Seoul, Korea

**Keywords:** Glycogen storage disease, Molecular diagnosis, *PYGL*, Next-generation sequencing

## Abstract

**Background:**

Glycogen storage disease type VI (GSD VI) is a rare disease in which liver glycogen metabolism is impaired by mutations in the glycogen phosphorylase L (*PYGL*). This study aimed to examine the clinical features, genetic analyses, and long-term outcomes of patients with GSD VI in Korea.

**Methods:**

From January 2002 to November 2022, we retrospectively reviewed patients diagnosed with GSD VI using a gene panel at Seoul National University Hospital. We investigated the clinical profile, liver histology, molecular diagnosis, and long-term outcomes of patients with GSD VI.

**Results:**

Five patients were included in the study. The age at onset was 18–30 months (median, 21 months), and current age was 3.7–17 years (median, 11 years). All patients showed hepatomegaly, elevated liver transaminase activity, and hypertriglyceridaemia. Hypercholesterolaemia and fasting hypoglycaemia occurred in 60% and 40% of patients, respectively. Ten variants of *PYGL* were identified, of which six were novel: five missense (p.[Gly607Val], p.[Leu445Pro], p.[Gly695Glu], p.[Val828Gly], p.[Tyr158His]), and one frameshift (p.[Arg67AlafsTer34]). All patients were treated with a high-protein diet, and four also received corn starch. All patients showed improved liver function tests, hypertriglyceridaemia, hepatomegaly, and height z score.

**Conclusions:**

The GSD gene panel is a useful diagnostic tool for confirming the presence of GSD VI. Genetic heterogeneity was observed in all patients with GSD VI. Increased liver enzyme levels, hypertriglyceridaemia, and height z score in patients with GSD VI improved during long-term follow-up.

**Supplementary Information:**

The online version contains supplementary material available at 10.1186/s13023-023-02750-1.

## Background

Glycogen storage disease type VI (GSD VI; OMIM #232700) is an inborn error of glycogen metabolism caused by mutation of the hepatic glycogen phosphorylase L (*PYGL*) gene located on chromosome 14q21-q22 with an autosomal recessive inheritance [1]. As a result, *PYGL* deficiency occurs, which is involved in the rate-limiting step of glycogen degradation [2]. GSD VI was first reported by Henry-Gery Hers in 1959, and its estimated prevalence was 1 per 100,000 person-years [1,  3].

The onset of GSD VI is usually infancy or early childhood, with typical clinical manifestations including hepatomegaly, growth retardation, ketotic hypoglycaemia, elevated hepatic transaminases, and hypertriglyceridemia [4]. Because gluconeogenesis is intact, hypoglycaemia is relatively less severe compared to GSD I, but some patients experience significant life-threatening hypoglycaemia [5]. Cardiomyopathy, focal nodular hyperplasia, hepatocellular carcinoma, and abnormal bone mineralisation have also been reported in patients with GSD VI [5–8].

The gold standard for the diagnosis of GSD VI is enzyme testing on a liver biopsy, but it is invasive, so the latest guidelines recommend molecular diagnosis first [1]. In addition, clinical manifestations such as hepatomegaly and hypoglycaemia are also present in other types of GSD. In particular, the clinical phenotype of GSD VI overlaps with that of GSD IX, which can be distinguished only by molecular diagnosis [4, 9].

No studies have examined the clinical features and *PYGL* variants of patients with GSD VI in Korea. Thus, this study aimed to investigate the clinical features, liver histology, *PYGL* variants, and long-term outcomes of patients with GSD VI in Korea.

## Results

### Initial clinical features of patients with *PYGL* variants

Four pathogenic and six likely pathogenic variants in *PYGL* were identified in 5 patients with GSD VI, and none had a family history of the disease. The clinical features of the patients with GSD VI are shown in Table 1 and Additional file 1. There were three male and two female patients currently aged 3.7–17 years (median, 11 years). The age at onset was 1.5–2.5 years (median, 1.9 years). The median age at diagnosis was 3.3 years (range, 1.8–6.0 years). The initial clinical symptoms were hepatomegaly and distended abdomen in five patients, but there were no cases of short stature, splenomegaly, or cardiomyopathy. One patient showed developmental delays and was eventually diagnosed with autism. All five patients showed elevated liver transaminase activity, hypertriglyceridaemia, and hypovitaminosis D. Hypercholesterolaemia, postprandial hyperlactatemia, and fasting hypoglycaemia occurred in 60%, 60% and 40% of the patients, respectively. One patient had hypercalciuria with a relatively small unilateral kidney on ultrasonography.

**Table 1:** Clinical and histologic features of patients with glycogen storage disease VI

Clinical data	Case 1	Case 2	Case 3^a^	Case 4	Case 5
Sex	M	M	F	M	F
Age of onset (years)	2.0	1.5	2.5	1.8	1.7
Age of diagnosis (years)	3.3	3.5	2.6	1.8	6.0
Presenting symptoms	Abdomen distention	Abdomen distention, Developmental delay	Abdomen distention, AST/ALT elevation	Abdomen distention	Abdomen distention
AST/ALT (U/L)^b^	203/324	216/362	387/458	279/179	133/117
Uric acid (mg/dL)^b^	3.8	5.1	3.9	4.7	3.0
Cholesterol (mg/dL)^b^	99	177	112	185	201
TG (mg/dL)^b^	173	139	237	106	188
Lactic acid (mmol/L)^b^	2.9	6.3	2	1.2	2.5
Glucose (mg/dL) (fasting)^b^	75	82	50	59	74
Age at liver biopsy (years)	3.3	3.5	2.5	1.8	2.8
Light microscope	Periportal and septal fibrosis	Periportal, perisinusoidal, and perivenular fibrosis	Periportal, perivenular, and pericellular fibrosis	Periportal fibrosis	Periportal fibrosis
Electron microscope	Swollen hepatocytes with glycogen deposits, lipid vacuoles, and collagen deposits	Swollen hepatocytes with glycogen deposits	Swollen hepatocytes with glycogen deposits and lipid vacuoles	Swollen hepatocytes with glycogen deposits, lipid vacuoles, and collagen deposits	Swollen hepatocytes with glycogen deposits and lipid vacuoles
Treatment	Corn starch (4–6 g/kg/day), high protein diet (2 g/kg/day)	Corn starch (6–8 g/kg/day), high protein diet (3 g/kg/day)	Corn starch (4–6 g/kg/day), high protein diet (2-2.5 g/kg/day)	Corn starch (8 g/kg/day), high protein diet (2 g/kg/day)	High protein diet (2.5 g/kg/day)

^a^Case 3 had a small unilateral kidney on ultrasonography

^b^Lab parameters were collected at age of diagnosis. AST: aspartate transaminase; ALT: alanine transaminase; TG: triglyceride; NA: not available

### Liver histology

**Fig. 1:**
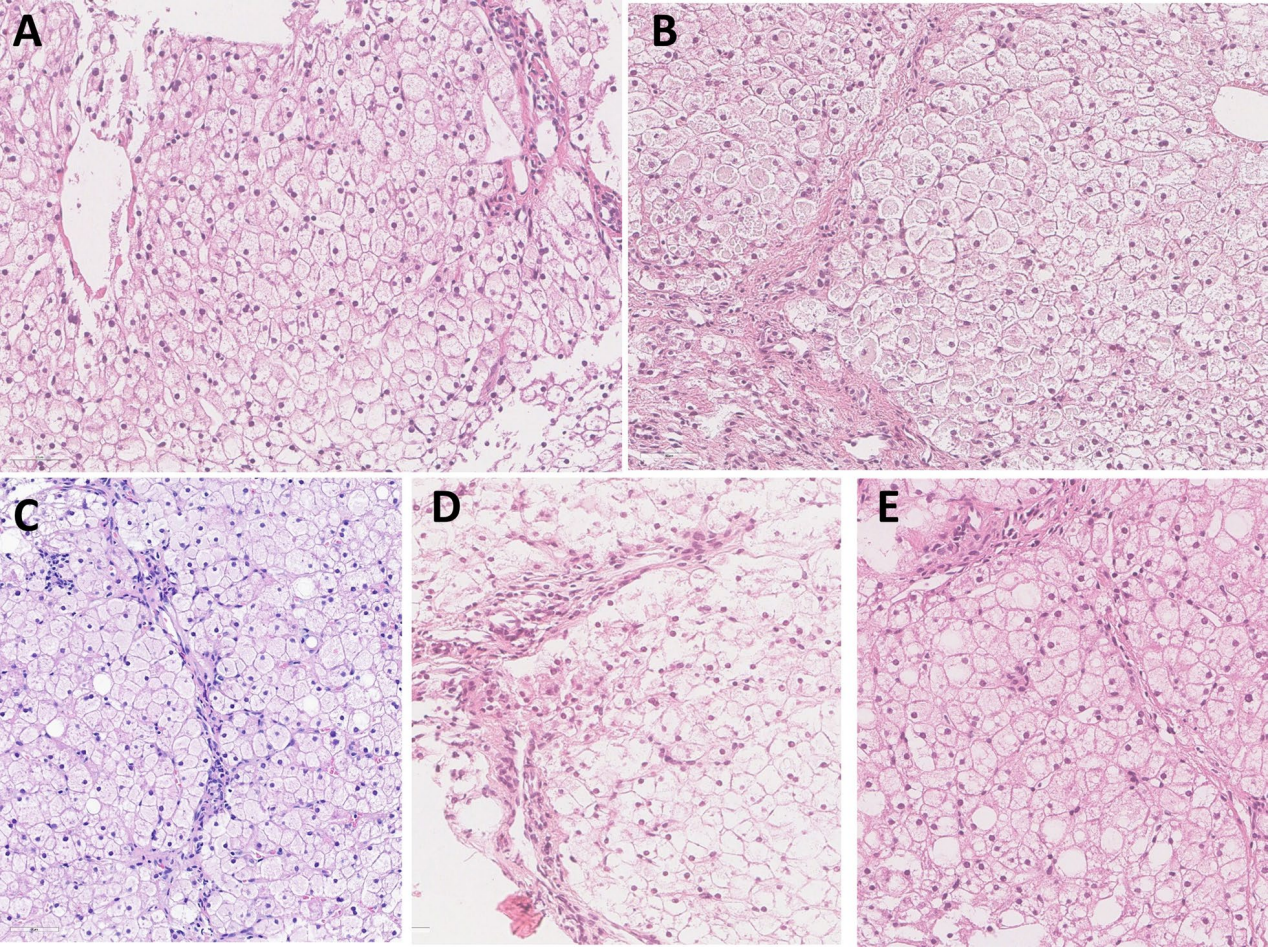
Representative histological findings from five patients with GSD VI. Regardless of patients, histological findings were similar with swollen hepatocytes, invisible sinusoids, and slender periportal fibrosis (x400) (**A**, Case 1; **B**, Case 2; **C**, Case 3; **D**, Case 4; **E**, Case 5)

The age at liver biopsy were 1.8–3.5 years. All patients had enlarged hepatocytes with glycogenated nuclei and periportal fibrosis. Perivenular fibrosis was identified in two (40%) patients, while perisinusoidal, pericellular, and septal fibrosis was identified in one (20%) patient, respectively. Two (40%) patients had hepatic steatosis, two (40%) showed mild portal and lobular inflammation, and none showed cirrhosis. Electron microscopy revealed swollen hepatocytes with glycogen deposits in all patients, lipid vacuoles in 80% of patients, and collagen deposits in 20% of patients (Fig. 1).

### Molecular genetic analysis

Ten variants of the *PYGL* gene in patients with GSD VI were identified in this study: six missense variants, c.1820G > T p.(Gly607Val), c.1727G > A p.(Arg576Gln), c.1334T > C p.(Leu445Pro), c.2084G > A p.(Gly695Glu), c.2483T > G p.(Val828Gly), and c.472T > C p.(Tyr158His); two nonsense variants, c.280 C > T p.(Arg94Ter) and c.2446 C > T p.(Arg816Ter); and one splice variant, c.1768 + 1G > A; one frameshift variant, c.198delG p.(Arg67AlafsTer34) (Table 2). Genetic heterogeneity was observed in all patients with GSD VI. Six variants were novel, not observed in the normal population, and located at highly conserved loci in various species: p.(Gly607Val), p.(Arg67AlafsTer34), p.(Leu445Pro), p.(Gly695Glu), p.(Val828Gly), and p.(Tyr158His). The novel variants identified in this study were registered in the Clinvar database. All variants were considered pathogenic or likely pathogenic according to the American College of Medical Genetics and Genomics (ACMG) classification.[10].

**Table 2:** Genetic analysis of 5 patients with glycogen storage diseases VI in our study

Case	cDNA change	Amino acid change	Variant type	ACMG classification
1	c.1768 + 1G > A **c.1820G > T**	**NA ** **p.(Gly607Val)**	Splicing Missense	P LP
2	**c.198delG** c.1727G > A	**p.(Arg67AlafsTer34)** p.(Arg576Gln)	Frameshift Missense	P LP
3	**c.1334T > C** **c.2084G > A**	**p.(Leu445Pro)** **p.(Gly695Glu)**	Missense Missense	LP LP
4	c.280 C > T **c.2483T > G**	p.(Arg94Ter) **p.(Val828Gly)**	Nonsense Missense	P LP
5	c.2446 C > T **c.472T > C**	p.(Arg816Ter) **p.(Tyr158His)**	Nonsense Missense	P LP

ACMG: American College of Medical Genetics and Genomics; P: pathogenic; LP: likely pathogenic; VUS: variant of uncertain significance; NA: not available

In bold indicate a novel variants

### Long-term outcomes of patients with *PYGL* variants

**Fig. 2:**
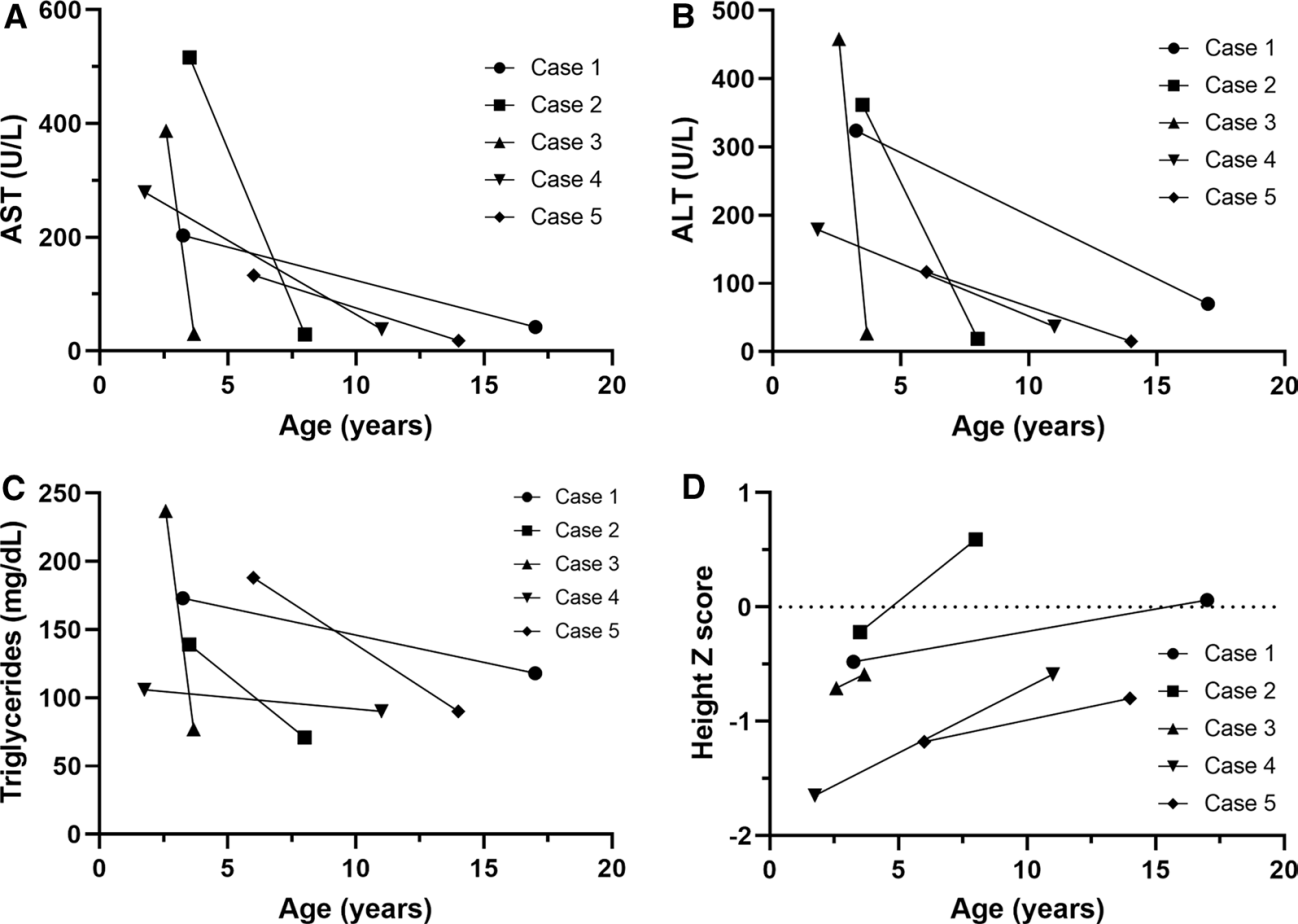
Long term follow up of biochemical and growth parameters. **A** Serum AST levels; **B** Serum ALT levels; **C** Serum triglycerides levels; **D** Height z score of patients in this study were compared baseline and follow-up values; AST, aspartate aminotransferase; ALT, alanine aminotransferase

The age range at last follow up were 3.7 to 17.0 years (median, 11 years), and the patients’ follow-up durations were 12 months to 14.5 years (median, 8 years). Once the diagnosis was confirmed, all patients received a high-protein diet during the follow-up period, and four also consumed corn starch. The levels of aspartate transaminase, alanine transferase, and triglycerides and height z score of the patients significantly improved after the long-term follow-up, as shown in Fig. 2 (P < 0.05). In addition, hepatomegaly improved in all patients on physical examination and ultrasonography. None of the patients developed hepatic adenomas or underwent liver transplantation.

## Discussion

Here we investigated the spectrum of clinical features, liver histology, *PYGL* variants, and long-term outcomes of patients with GSD VI in Korea. All patients with GSD VI showed hepatomegaly, a distended abdomen, elevated liver transaminases, and hypertriglyceridaemia at the initial presentation; all improved in the long-term follow-up with supplementation of a high-protein diet and corn starch. All patients showed enlarged hepatocytes and periportal fibrosis on liver biopsy. Ten *PYGL* variants were identified, of which six were novel.

In our study cohort, the median age at diagnosis was 3.3 years old, comparable with other studies.[11, 12] Case 5 showed abdominal distention at 1.7 years of age but was diagnosed at 6 years of age, suggesting that the diagnosis was considerably delayed. Other studies reported a delayed diagnosis[12] related to the disease characteristics of GSD VI. As gluconeogenesis is intact in patients with GSD VI, they show less severe symptoms compared to other types of GSD, and the diagnosis may be delayed. The most important symptom in this study was a distended abdomen with hepatomegaly, similar to several other studies.[11, 13, 14] However, many studies reported patients with short stature versus none in our study.[11, 13, 15] Case 2 showed developmental delay and was eventually diagnosed with autism. Other studies reported developmental delay or muscle hypotonia in patients with GSD VI[16, 17] and autism in patients with GSD II,[18] but no studies have reported autism in patients with GSD VI.

All patients in this study showed elevated liver transaminases and hypertriglyceridaemia, while some patients showed fasting hypoglycaemia and postprandial hyperlactatemia, consistent with the findings of other studies.[4, 11, 13, 17, 19, 20] All patients in this study showed hypovitaminosis D at the initial presentation, and other studies reported that abnormal bone mineralisation and osteopenia or osteoporosis may appear as complications of GSD VI.[2, 8] Therefore, monitoring of 25-OH vitamin D levels and regular bone mineral density tests are recommended. Hypercalciuria was identified and improved with age in one patient with GSD VI in our study cohort. Hypercalciuria has been reported in patients with GSD I[21, 22]; however, this is the first report of hypercalciuria in patients with GSD VI. Based on our findings, urine samples should be assessed in patients with GSD VI at the time of diagnosis. A study reported in Canada showed mild cardiomyopathy in one patient with GSD VI[5], whereas no patients in our study cohort had cardiomyopathy.

Enlarged hepatocytes with glycogenated nuclei and liver fibrosis were identified in all patients with GSD VI in this study, consistent with other studies.[1, 2, 5, 13, 15] Studies have reported hepatocellular carcinoma or liver cirrhosis[4, 6], but neither was identified in patients in this study. Hepatic steatosis, lobular inflammation, and lipid vacuoles have been reported in patients with GSD VI as in this study.[13, 15].

To date, approximately 106 patients with GSD VI[5, 11–14, 17, 23, 24] and about 90 pathogenic *PYGL* variants have been reported[11]. Those in our study (c.1768 + 1G > A, p.[Arg576Gln], p.[Arg94Ter], and p.[Arg816Ter]) were reported in Israel[23], China[11, 15], and Switzerland[12], respectively. We identified six novel variants of *PYGL* in this study that were not observed in the normal population and were located at highly conserved loci in various species: p.(Gly607Val), p.(Arg67AlafsTer34), p.(Leu445Pro), p.(Gly695Glu), p.(Val828Gly), and p.(Tyr158His). A founder pathogenic variant of c.1620 + 1G > A was reported in the USA[25]; and c.777T > A, p.(Asn259Lys), and c.1900G > C, p.(Asp634His) were frequently reported in patients in Switzerland.[12] In addition, high-frequency variants of c.1621 − 258_2178-23 del, c.1621 − 258_2178-26 del, c.2467 C > T, p.(Gln823Ter), and c.772 + 1G > A were reported by a study in China,[11, 14, 15] while c.2071G > C, p.(Gly691Arg), and c.345G > A were frequently reported in patients with GSD VI in England [13] and Turkey,[19] respectively. In this study, common variants were not found, and genetic heterogeneity was observed, which was similar to a French study reporting no mutational hotspot.[20] The most common pathogenic variant was missense (60%), similar to the results of other studies.[4, 11, 17] Due to the diversity of genetic variants in this study cohort, genotype–phenotype correlations were difficult to assess.

In this study, patients with GSD VI received a high-protein diet and corn starch. The recommended daily amount of a high-protein diet is 2–3 g/kg, while that of corn starch is 5–8 g/kg. Hepatomegaly, elevated liver enzymes, increased triglyceride levels, and height z score of the patients in this study significantly improved after long-term follow-up, consistent with other studies.[11, 13, 15, 19, 26] As a patient with GSD VI lacks phosphorylase and uses protein substrates for gluconeogenesis to compensate for low glucose production, a high-protein diet enriches protein substrates, aiding gluconeogenesis.[1, 27].

This study has several limitations. First, the maximum age at follow-up among the patients with GSD VI in this study was 17.0 years old. Although there were no patients with GSD VI with HCC or liver cirrhosis in this study, the age of the patient with GSD VI with HCC in the literature was 23 years old.[6] Therefore, regular follow-up is needed as HCC or decompensated liver cirrhosis may develop in these patients in the future. Second, the number of patients with GSD VI was small and the study was conducted at a single centre. A multicentre or multinational study involving a large number of patients with GSD VI is required to validate our findings.

## Conclusions

Our study reported the clinical features, liver histology, genetic analysis, and long-term outcomes of patients with GSD VI in Korea. Increased liver enzyme levels, hypertriglyceridaemia, and height z score in patients with GSD VI improved in the long-term follow-up. The GSD gene panel is a useful diagnostic tool for confirming the presence of GSD VI. Genetic heterogeneity was observed in all patients with GSD VI.

## Methods

### Study population and data collection

The cases of all patients with GSD VI at Seoul National University Children’s Hospital treated between January 2002 and November 2022 were retrospectively reviewed. The diagnosis of GSD VI was based on excessive glycogen accumulation in a liver biopsy specimen and the identification of pathogenic or likely pathogenic variants in *PYGL* gene using a GSD gene panel. Clinical phenotypes, laboratory findings, pathology reports, and long-term outcomes were reviewed in the patients’ electronic medical records.

Fasting hypoglycaemia was defined as a blood glucose level < 70 mg/dL for more than 4 h after meals, while hypertriglyceridaemia and hypercholesterolaemia were defined as higher than normal blood levels according to age.[28, 29] Hyperlactatemia was defined as a blood lactic acid levels more than 2.3 mmol/L after meals. Hypercalciuria was defined as a random urine calcium to creatinine ratio > 0.2.

### Genetic test of *PYGL*

The GSD gene panel contained *AGL, G6PC, GBE1, GYS2, PHKA2, PHKB, PHKG2, PYGL, SLC2A2*, and *SLC37A4*, and NM_002863 was used for reference transcript for *PYGL*. Pre-capture libraries (Illumina, Inc., San Diego, CA, USA) and capture processes (Agilent Technologies, Santa Clara, CA, USA) were performed according to the manufacturer’s protocol. The captured libraries were sequenced using MiSeqDx (Illumina, Inc., San Diego, CA, USA). The raw sequence data were analysed using NextGENe software (SoftGenetics, State College, PA, USA) and annotated with ANNOVAR (http://annovar.openbioinformatics.org). Common variants were filtered using the gnomAD (http://gnomad.broadinstitute.org) and KRG (http://coda.nih.go.kr/coda/KRGDB) databases. The Human Gene Mutation Database and ClinVar were used to identify known pathogenic variants. The sequence variant was evaluated with a computational (in silico) predictive program using PolyPhen-2, SIFT, and MutationTaster. The pathogenicity of sequence variants was evaluated using the 2015 ACMG guidelines [10].

### Statistical analyses

The clinical and laboratory findings of patients with a molecular genetic diagnosis were statistically analysed. SPSS for Windows (version 25; IBM Corp, Armonk, New York) software was used to perform the statistical analyses. A paired t-test was performed to compare the clinical characteristics at baseline and follow-up in the same patients. Statistical significance was set at P < 0.05.

## Supplementary Information

**Additional file 1.** Clinical vignette of  patients with glycogen storage disease VI.

## Data Availability

All data generated or analysed during the current study are available in the clinVAR repository (https://www.ncbi.nlm.nih.gov/clinvar; accession Nos. SCV002754424-SCV002754429).

## Abbreviations

GSD: Glycogen storage disease
PYGL: Glycogen phosphorylase L
