# Supplementary material for: Clinical and genetic spectrum of GSD type 6 in Korea
Source: Orphanet J Rare Dis. 2023 Jun 1;18:132. doi: 10.1186/s13023-023-02750-1 (PMC10233917; doi:10.1186/s13023-023-02750-1)
Supplement: Supplementary file 1 — Additional file 1. Clinical vignette of patients with glycogen storage disease VI. [file 13023_2023_2750_MOESM1_ESM.docx]

**Supplementary Appendix**

**Clinical and genetic spectrum of GSD type 6 in Korea**

　 　 Jong Woo Hahn, MD^1,2^, Heerah Lee, MD^3^, Moon Woo Seong, MD, PhD^3^, Gyeong Hoon Kang, MD, PhD^4^, Jin Soo Moon, MD, PhD^1^, and Jae Sung Ko, MD, PhD^1*^

^1^Department of Pediatrics, Seoul National University College of Medicine, Seoul, Korea
^2^Department of Pediatrics, Seoul National University Bundang Hospital, Seongnam, Gyeonggi-do, Korea
^3^Department of Laboratory, Seoul National University College of Medicine, Seoul, Korea
^4^Department of Pathology, Seoul National University College of Medicine, Seoul, Korea

**Correspondence**

Jae Sung Ko, MD, PhD

Professor

Division of Pediatric Gastroenterology, Hepatology and Nutrition, Department of Pediatrics

Seoul National University College of Medicine

Seoul National University Children's Hospital

101 Daehak-ro, Jongno-Gu, Seoul, 03080, Korea

President, The Korean Society of Pediatric Gastroenterology, Hepatology, and Nutrition

Vice Chairman, The Korean Society of Gastroenterology

Tel; 82-2-2072-2197, Fax; 82-2-743-3455

E-mail : [kojs@snu.ac.kr](mailto:kojs@snu.ac.kr)

**SUPPLEMENTARY RESULTS**

Clinical vignette 1:

A 39-month-old boy was referred to our hospital for further evaluation of hepatomegaly and elevation of liver transaminase. Abdominal distention occurred at 2 years of age, and there was no specific findings in the family history. On physical examination, the liver was palpable 4 finger breadths below the right costal margin. His height was 95 cm (z score -0.48) and body weight was 14.4kg (z score -0.23). The laboratory examination revealed that aspartate transaminase (AST) 203 U/L, alanine transaminase (ALT) 324 U/L, triglyceride 173 mg/dL, 25(OH) vitamin D 10.7 ng/ml, and postprandial lactic acid 2.9 mmol/L. Liver biopsy was performed, and light microscopy showed large hepatocytes with prominent cell membranes and glycogenated nuclei, and periportal fibrosis was found. Electron microscopy showed that hepatocytes were swollen with glycogen particles and intracytoplasmic fat vacuoles. Genetic analysis was done and 1 pathogenic and 1 likely pathogenic variants of *PYGL* gene were identified. He treated with uncooked cornstarch (4-6g/kg/day) and high protein diet (2g/kg/day). He has been under outpatient follow-up for about 14.5 years, and hepatomegaly reduced to 2 finger breaths below the right costal margin. His height was 176.cm (z score 0.06) and body weight 64.4kg (z score -0.19), and laboratory results showed AST 42 U/L, ALT 70 U/L.

Clinical vignette 2:

A 42-month-old boy visited to our hospital for further evaluation of hepatomegaly. He had hepatomegaly since 18 months of age. Liver was palpable 2 finger breadths below the right costal margin. His height was 97.2cm (z score -0.22) and body weight was 11.7kg (z score -2.45). He had motor developmental delay. He was able to pull up to stand at 16 months and walk independently at 19 months of age. His language development was slightly delayed, but within the normal range. The laboratory results showed AST 516 U/L, ALT 362 U/L, cholesterol 177 mg/dL, TG 139 mg/dL, 25(OH) vitamin D 9.6 ng/ml, and postprandial lactic acid 6.3 mmol/L. Liver biopsy showed diffuse enlargement of hepatocytes with periportal, perisinusoidal, and perivenular fibrosis in light microscopy. Intracytoplasmic pools of glycogen particles were observed in electron microscopy. One pathogenic and one likely pathogenic variants of *PYGL* gene were identified in genetic analysis. He treated with uncooked corn starch (6-8g/kg/day) and high protein diet (3g/kg/day). He has been under outpatient follow-up for about 3.5 years. The last physical examination on liver showed 1 finger breadths below the right costal margin. His height was 120cm (z score 0.59) and body weight was 20kg (z score -0.45), and levels of AST and ALT improved to 29 U/L and 19 U/L, respectively. He was diagnosed with autism, eventually.

Clinical vignette 3:

A 31-month-old girl was referred to our hospital for hepatomegaly and elevation of liver transaminase. Abdomen distention occurred at 30 months of age. On physical examination, liver was palpable 4 finger breadths below the right costal margin. Her height was 88cm (z score -0.71) and body weight was 12.4kg (z score -0.51). The levels of AST was 387 U/L, ALT 458 U/L, TG 237 mg/dL, and 25(OH) vitamin D 13.9 ng/mL. She showed fasting hypoglycemia and hypercalciuria with a relatively small unilateral kidney on ultrasonography. Liver biopsy was performed, and light microscopy showed enlarged hepatocytes with periportal, perivenular, and pericellular fibrosis. Electron microscopy showed swollen hepatocytes with glycogen deposits and lipid vacuoles. Genetic analysis identified two likely pathogenic variants of *PYGL*. She treated with corn starch (4-6g/kg/day) and high protein diet (2-2.5g/kg/day). For 12 months of follow-up visit, the liver size decreased to 2 finger breadths below the right costal margin, and liver transaminases returned to normal. The z score of her height and body weight were improved.

Clinical vignette 4:

A 21-month-old boy visited to our hospital for further investigation of hepatomegaly. He had hepatomegaly since 21 months of age. Liver was palpable 4 finger breadths below the right costal margin. His height was 80.4cm (z score -1.65) and body weight was 11.6kg (z score 0.04). The laboratory results showed AST 279 U/L, ALT 179 U/L, cholesterol 185 mg/dL, and 25 (OH) vitamin D 14.1 ng/ml. The blood glucose level was reduced to 59 mg/dL after fasting. Liver biopsy showed diffuse swelling of hepatocytes with periportal fibrosis in light microscopy. Hepatocytes with glycogen deposits, lipid vacuoles, and collagen deposits were observed in electron microscopy. One pathogenic and one likely pathogenic variants of *PYGL* were identified in genetic analysis. He treated with corn starch (8g/kg/day) and high protein diet (2g/kg/day). He was under outpatient follow-up for about 9 years, the last physical examination on liver showed 2 finger breadths below the right costal margin. The z score of his height and body weight were improved. (-0.59, 1.04, respectively), and levels of AST and ALT returned to normal values.

Clinical vignette 5:

A 6-year-old girl was referred to our hospital for hepatomegaly. Abdomen distention occurred at 20 months of age. On physical examination, liver was palpable 4 finger breadths below the right costal margin. Her height was 105.5cm (z score -1.18) and body weight was 17.8kg (z score -0.49). The levels of AST was 133 U/L, ALT 117 U/L, cholesterol 201 mg/dL, TG 188 mg/dL, 25 (OH) vitamin D 11.5 ng/ml, and postprandial lactic acid 2.5 mmol/L. Liver biopsy was performed and light microscopy showed enlarged hepatocytes with periportal fibrosis. Electron microscopy showed hepatocytes with glycogen deposits and lipid vacuoles. Genetic analysis identified one pathogenic and one likely pathogenic variants of *PYGL*. She treated with high protein diet (2.5g/kg/day) only. She has been under outpatient follow-up about 8 years, and hepatomegaly was reduced to 2 finger breaths below the right costal margin at the last follow-up. Liver transaminases returned to normal and her height was 154cm and body weight was 41.1kg (z score -0.8 and -0.88, respectively).
